# Relationship between Cardiorespiratory Fitness and Cardiovascular Diseases Risk Factors in Iranian Children

**Published:** 2012-07-30

**Authors:** N Kazemi, F Movaseghi, M Koshki

**Affiliations:** 1 Department of Physical Education, Science and Research Branch, Islamic Azad University, Fars, Iran; 2 Department of Physical Education, Sepidan Branch, Islamic Azad University, Fars, Iran; 3 Department of Physical Education, Shiraz University, Shiraz, Iran

**Keywords:** Cardiorespiratory fitness, Cardiovascular risk factors, Children, Iran

Dear Editor,

Cardiovascular Diseases (CVD) are the leading cause of global mortality, accounting for almost 17 million deaths annually.[1] Therefore, CVD is responsible for a substantial amount of money spent by health care systems for its treatment, and treatment of its symptoms. Although, CVD events occur most frequently during or after the fifth decade of life, there are evidences indicating that the precursors of CVD have origins in childhood.[2] In addition, adverse CVD risk factors during childhood have been shown to track into adulthood.[3] One of the most recognized CVD risk factor in children is the cardiorespiratory fitness,[4] which influences the cardiovascular health in children and adolescents. However, the association between cardiorespiratory fitness and cardiovascular risk factors in children has remained unclear. Therefore, the aim of this study was to evaluate the relationship between cardiorespiratory fitness with cardiovascular disease risk factors in 8 to 12 years old children in southern Iran.

A total of 132 children at an elementary school in Noorabad, with the age of 9.73±1.38 years, standing height of 134.5±11.77 cm, body weight 32.04±8.65 kg and body mass index 17.46±2.58 who were volunteer to participate in this study were enrolled. They were informed about the study and an informed consent was provided from each participant.

Standing height was measured to the nearest 1 mm using a wall mounted stadiometer. Weight was measured using an electronic scale to the nearest 0.1 kg. Body Mass Index (BMI) was calculated as participants’ weight in kilograms divided by the square of their height in meters (Kg/m^2^). Body fat percentage (BF%) was calculated by Jackson formula:

BF%=(1.51×BMI)-(0.7×Age)-(3.6×Sex)+1.4

Both systolic and diastolic blood pressures were taken using an automated sphygmomanometer and a children-sized cuff. For blood collection, following an overnight fasting, 5 ml intravenous blood sample was taken from the children’s antecubital vein. Standard enzymatic methods were used for measuring levels of serum total cholesterol, triglycerides, high-density lipoprotein cholesterol (HDL-C) and glucose levels. Low-density lipoprotein cholesterol (LDL-C) concentration was calculated by the Friedewald formula [LDL=TC-HDL-T.G/5(mg/dl)].

Cardiovascular fitness was determined as treadmill time to exhaustion by Bruce protocol. The length of the time that each child could tolerate on running treadmill was the test score and used to estimate the VO_2max_: VO_2max_=14/76-1/379(T)+0/451(T)-0/012(T).

Linear regression model of the relationships between cardiorespiratory fitness and cardiovascular disease risk factors in children were presented in Table 1, which shows that high levels of cardiorespiratory fitness were inversely associated with BMI, BF%, TG and LDL. Therefore, higher cardiorespiratory fitness is associated with a better lipid profile in children, and obesity as a cardiovascular risk factor can be most successfully influenced by improving cardiorespiratory fitness.

**Table 1:** Linear regression model of the relationships between cardiorespiratory fitness and cardiovascular disease risk factors in children.

**Variable**	**Regression coefficient**	**SE**	***P* value**
SBP	-0.003	0.021	0.877
BMIa	-0.169	0.077	0.030
BF%^a^	-0.336	0.109	0.004
FBS	-0.370	0.25	0.146
TG^a^	-1.59	0.824	0.05
TC	-0.895	0.842	0.294
HDL	0.117	0.297	0.196
TC/HDL	-0.010	0.021	0.639
LDL^a^	-1.006	0.330	0.003

^a^ P<0.05

It should be mentioned that our results were in agreement with other reports in the literature, for example, Ruiz et al. evaluated the associations between cardiorespiratory fitness and metabolic risk factors in 873 healthy children aged 9–10 years,[5] that showed that cardiorespiratory fitness was negatively associated with a clustering of metabolic risk factors.

The results of our study add supportive evidence to the body of knowledge suggesting that cardiorespiratory fitness in children is an important health marker, and children/adolescents with higher physical activity have a lower number of biological risk factors for CVD.
